# Gene presence–absence and evolutionary signals of positive selection associated with virulence divergence in Beauveria fungi

**DOI:** 10.1099/mgen.0.001758

**Published:** 2026-06-26

**Authors:** Teeratas Kijpornyongpan, Nuntanat Arnamnart, Papichaya Kwantong, Alongkorn Amnuaykanjanasin, Janet Jennifer Luangsa-ard, Noppol Kobmoo

**Affiliations:** 1 Department of Botany and Plant Pathology, Purdue University, West Lafayette, IN, USA; 2 National Center for Genetic Engineering and Biotechnology (BIOTEC), National Science and Technology Development Agency (NSTDA), Pathum Thani, Thailand

**Keywords:** *Beauveria*, effector-like proteins, gene presence–absence variation, non-ribosomal peptide synthase (NRPS), positive selection, virulence

## Abstract

*Beauveria bassiana* is widely used as a mycoinsecticide and studied for its pathogenic mechanisms. However, the virulence biology of other *Beauveria* species remains poorly understood. In this study, we conducted comparative pathological and genomic analyses from seven species representing the *B. bassiana* and *Beauveria asiatica* species complexes. Virulence assays on *Spodoptera exigua* revealed substantial intra- and interspecific variability, with *Beauveria neobassiana* emerging as the most virulent. Notably, insect mortality does not correlate with ‘mycelia on cadavers’, highlighting the need for multiple virulence proxies. Draft genomes of the *Beauveria* species included in the virulence assays were assembled and annotated. Gene presence–absence analyses identified 195 orthogroups potentially related to virulence, including genes encoding proteases, transporters and lipid-modifying enzymes. Over 70% of these orthogroups lacked functional annotations, consistent with lineage-specific or rapidly evolving virulence factors. Genome-wide scans for positive selection occurring in high-virulence strains revealed enrichment in genes with signal peptides, suggesting roles as effectors. *B. bassiana* NHJ13051 and *Beauveria thailandica* MY4824 exhibited high numbers of gene gains and positively selected genes, suggesting independent evolution of virulence in different lineages. A secondary metabolite biosynthetic gene cluster containing terpene and non-ribosomal peptide synthase (NRPS) core genes is strongly associated with high-virulence strains. Targeted comparative analyses further showed that *B. neobassiana* harboured more NRPS core genes under positive selection than other *Beauveria* species included in this study. These findings reveal that virulence variation in *Beauveria* is shaped by lineage-specific gene gains, selection on secreted proteins and metabolic diversification. Our study provides a genomic framework for understanding entomopathogenicity beyond *B. bassiana* and highlights promising candidate genes for biocontrol enhancement.

**Impact Statement**

The entomopathogenic fungus *Beauveria bassiana* has been widely recognized as an effective biocontrol agent for insect pest management. While the molecular mechanism underlying virulence in this species has been extensively studied, the factors explaining interspecific variation within the genus *Beauveria* remain poorly understood. Here, we present one of the first investigations into genomic signatures linked to insect virulence at a multispecies scale. Our findings demonstrate substantial variation in virulence both within and between species. Through comparative genomic analyses of 16 fungal strains spanning 7 *Beauveria* species, we identified a focused set of candidate genes potentially involved in virulence. These candidates offer promising targets for further functional characterization and could be leveraged to improve biocontrol efficacy. The reference genomes generated from this study will be valuable resources for conducting future comparative analyses to understand the evolution of insect pathogenesis in the genus *Beauveria*.

## Data Summary

The genome assembly and annotation of the 15 *Beauveria* genomes are deposited at GenBank under the BioProject accession PRJNA744643 and genome accessions JBRFMC000000000–JBRFMP000000000 and JBXUMC000000000. MGI2000 sequencing raw reads used in this study are from a previous study [1]. The gene prediction data of *Beauveria neobassiana* BCC2660, as well as the functional annotation data from eggNOG-mapper, InterProScan, db-CAN and antiSMASH is available in the FigShare data repository under the following link https://doi.org/10.6084/m9.figshare.30157447.

## Introduction

*Beauveria* (*Cordycipitaceae*, *Ascomycota*) is a cosmopolitan fungal genus of entomopathogens. It is considered a facultative pathogen as it can have a free-living stage as a soil inhabitant or a plant endophyte [2,3]. A member of this genus, *Beauveria bassiana*, is one of the most widely used fungi as a mycoinsecticide for the last two decades due to its efficacy in killing insect pests and its fast growth and sporulation [3]. Continuous research and development efforts have focused on screening *B. bassiana* strains [4,7] as well as the emergence of new formulation strategies for preparing inoculum [3].

*Beauveria bassiana* infects and colonizes insect hosts through multiple steps, including an initial attachment of spores on the host’s cuticle, spore germination and penetration into the insect’s body, vegetative growth in host hemocoel, complete colonization and eventually sporulation [3,8, 9]. Previous molecular genetic studies have elucidated genes affecting virulence [10]. These include hydrophobin genes for host attachment, as well as chitinase and subtilisin genes for cuticle degradation [11,14]. Secondary metabolite biosynthetic genes such as polyketide synthase (PKS) and non-ribosomal peptide synthase (NRPS) genes are crucial for producing host-killing toxins, e.g. beauvericin and bassianolide [15,17]. Several other genes associated with various functions such as transcription factors, signal transductions and transporters were also proposed [10]. Recent advancements in transcriptomics, proteomics and metabolomics also facilitate the discovery of additional virulence factors that may influence the pathogenesis of *B. bassiana* [8]. However, little is known about the pathogenesis of other *Beauveria* species, which are also entomopathogens on various types of insect hosts.

Despite the widespread use of *B. bassiana* in agriculture and biocontrol, taxonomic study of the genus *Beauveria* is often difficult due to morphological similarity [1,2, 5]. The species identification relies principally on multigene phylogenies [2,5, 18] because usual DNA barcodes such as internal transcribed spacers (*ITS*) or elongation factor 1-alpha (*TEF*) lack sufficient resolution to discriminate between closely related species. This impedes accurate species identification and better understanding of pathogen evolution, as well as the exploitation of *Beauveria* biodiversity [2,3]. Our previous study utilized genome-scale datasets for resolving *B. bassiana* and *Beauveria asiatica* species complexes, which proposed the nomination of three new species: *Beauveria thailandica*, *Beauveria namnaoensis* and *Beauveria neobassiana* [1]. This hidden diversity may underlie important variation in virulence traits and ecological adaptation among *Beauveria* species.

The first reference genome for the genus *Beauveria* was published by Xiao *et al*. [19], for *B. bassiana* ARSEF2860. Subsequent studies have produced additional reference genomes for *B. bassiana*, enabling comparative analyses to identify genes potentially involved in virulence. For example, Valero-Jiménez *et al*. [20] found unique toxin-producing genes, including NRPS, PKS and bacterial-like toxins in the hypervirulent strain *B. bassiana* Bb8028. Zhang *et al*. [21] compared 17 *B. bassiana* strains and identified hypermutated genes under positive selection such as NRPS and genes involved in a two-component signalling pathway that regulates fungal toxicity towards insects. A dual pathological and genomic study by Gasmi *et al*. [22] found that a number of non-synonymous changes in a chitinase are correlated with *B. bassiana* virulence against *Tenebrio molitor* and *Bemisia tabaci*.

Beyond *B. bassiana*, reference genomes have been generated for *Beauveria brongniartii*, *Beauveria pseudobassiana* and *B. neobassiana* [23,25]. However, many studies in the literature focused on the intraspecific variation of virulence and genomic features in *B. bassiana*, while multispecies comparative pathology and genomics to discover virulence-associated genes have rarely been conducted for the genus *Beauveria*. Our recent genomics-based taxonomic elucidation of *Beauveria* [1] now offers a unique opportunity to investigate the molecular mechanisms underlying interspecific variation in virulence at a variable scale, either between closely related species (within a species complex) or between more distantly related species (between species complexes).

In this study, we performed comparative genomics analyses combined with virulence assays on beet armyworms (*Spodoptera exigua*, *Lepidoptera*) to identify novel candidate genes potentially associated with virulence in *Beauveria*. Sixteen *Beauveria* isolates representing seven species across multiple phylogenetic depths (*B. asiatica*, *B. bassiana*, *B. gryllotalpidicola*, *B. mimosiformis*, *B. namnaoensis*, *B. neobassiana* and *B. thailandica*) were included. Fifteen *de novo* draft genomes were generated, complementing the available reference genome of *B. neobassiana* BCC2660 [25]. Multiple approaches, including heuristic scoring of gene presence–absence patterns, detection of positive selection and mapping to differentially expressed genes under conditions in response to insect cues [26], were implemented. In addition to the genome-wide analyses, we selected genes hypothesized to contribute to virulence, including those encoding chitin-specific carbohydrate-active enzymes (CAZymes) and NRPS biosynthetic enzymes, to examine their evolutionary patterns and potential association to virulence. Overall, we found that *B. neobassiana* was the most virulent species in our assays, potentially associated with the emergence of unique secondary metabolite gene clusters and rapid evolution of NRPS core genes. Comparative analyses revealed multiple candidate virulence-associated genes, including secreted hydrolytic enzymes, effector-like proteins, transporters and genes involved in secondary metabolism.

## Methods

### Fungal strains

Sixteen strains of *Beauveria* species (Table S1, available in the online Supplementary Material) were selected to represent a broad phylogenetic diversity within the genus, based on the genomics-based taxonomic framework of Kobmoo *et al*. [1]. The sampling included closely related species within *B. bassiana sensu lato* (*B. bassiana sensu stricto*, *B. namnaoensis* and *B. neobassiana*) and species belonging to *B. asiatica sensu lato* (*B. asiatica sensu stricto*, *B. thailandica*). Additionally, one strain of *B. mimosiformis*, which is closely related to *B. asiatica sensu lato* [18], and *B. gryllotalpidicola*, an early-diverging outgroup to all other taxa [27], were included. These strains were either isolated in previous studies and maintained at BIOTEC Culture Collection (BCC), Thailand, or obtained via Entomomopathogenic Fungal Culture Collections (ARSEF).

### Virulence assay

All 16 *Beauveria* strains (Table S1) were inoculated on potato dextrose agar (PDA) from a culture stock kept in glycerol at 4 °C. The PDA plates were incubated at room temperature (approx. 25 °C) between 7 and 30 days until sporulation depending on the growth rate of each strain. Spores were harvested in 0.2% Tween 80 solution, counted using a haemocytometer and adjusted to a concentration of 10^8^ spores/ml. Three microlitres of spore suspension were injected into a beet armyworm second-instar larva at the joint between the second and the third last abdomen segments using a micro-needle (Hamilton 7803-05) under a stereo microscope. Thirty worms, divided into three replicates of ten individuals, were tested per fungal strain. A blank suspension (0.2% Tween 80 without inoculum) was used as a negative control. The treated worms were transferred into a 12-well plate (one worm in each well), supplied with a feeding medium containing 13% (w/v) mung bean, 1% yeast extract, 0.3% ascorbic acid, 0.125% sorbic acid, 0.25% methyl paraben, 0.3% casein, 0.3% vitamin stock, 0.15% vitamin E, 0.05% choline chloride, 0.08% formalin and 1.2% agar (v/v). The plates were incubated at 25 °C, and the worms were monitored everyday up to 7 days. At each day post-inoculation, we measured two values as proxies for fungal virulence. The first value is ‘insect mortality’, determined by worm immobility and changed body colour to dark brown/black with or without external mycelial development. The second value is ‘mycelial colonization’, which only considers dead insects covered with fungal mycelia throughout the worm body. These two values were calculated as percentages of insects manifesting respective symptoms. They allow distinguishing between the fungal strains, not only for their ability to inflict death on the insects but also for their ability to grow outward and complete the life cycle with sporulation. In this study, the mycelial colonization is considered an ecologically relevant trait reflecting fungal persistence and potential for secondary transmission, which are collectively considered for successful pathogens in natural ecosystems [10,28]. Based on our virulence data (see in ‘Results’), only strains with both proxies ≥70% at day 7 post-inoculation were assigned as high-virulence strains.

### Whole-genome sequencing data

As 15 of the 16 strains did not have any genome assembly (Table S1), we thus retrieved their whole-genome shotgun sequencing data (MGI2000 platform) from our previous study [1], under the BioProject accession PRJNA744643. The sequencing reads of these strains (Table S1) were used for genome assembly and annotation as described below (see Genome assembly and annotation).

The genome assembly of *B. neobassiana* BCC2660 was already available [25] and was retrieved directly from the NCBI Assembly database under the accession GCA_002224115.1, BioProject accession PRJNA378127. We annotated the BCC2660 genome assembly and used it for downstream comparative analyses with the other 15 *Beauveria* strains. Additional genome assemblies of *Beauveria* spp. and other taxa from Cordycipitaceae were included for phylogenomic analyses: *B. bassiana* ARSEF2860 [19], *B. bassiana* ARSEF8028 [20], *B. brongniartii* RCEF3172 [24], *B. pseudobassiana* RGM2184 [23], *Cordyceps militaris* CM01 [29], *Isaria fumosorosea* ARSEF2679 [24], *Lecanicillium lecanii* RCEF1005 [24] and *Torrubiella hemipterigena* BCC1449 [30]. The genome of *Metarhizium acridum* (Clavicipitaceae) strain CQMa102 [31] was included as an outgroup.

### Genome assembly and annotation

We assembled the genome of the 15 *Beauveria* strains by applying the following procedure. First, sequencing reads were checked for quality using FastQC 0.12.1 [32]. The sequencing reads of all strains were adapter-free and passed base/sequence quality control. We then assembled the genome of each fungal strain using SPAdes 3.15.5 [33], by running a single paired-end library with the --careful mode and k-mers equal to 121. Quality assessment was conducted using Quast 5.2.0 and BUSCO 5.4.7 with reference to the orthologous set of Kingdom Fungi Odb10 [34,35]. Repeatmasker 4.1.2-p1 was used to soft-mask repetitive elements and low-complexity regions [36].

Next, we performed gene prediction and annotation for the 15 draft genomes of *Beauveria* spp., plus the draft genome of *B. neobassiana* BCC 2660. We first utilized GeneMark 4.69 for *ab initio* gene prediction with GeneMark-ES mode for fungi [37], followed by the gene prediction using MAKER 3.01.03 [38], which integrated three evidence sources: (1) an external prediction model in GFF format from GeneMark-ES; (2) a supervised-training prediction from Augustus 3.2.1, using the *Neurospora crassa* protein model as a training set [39]; and (3) external protein sequences from *B. bassiana* ARSEF2860 [19] for the blast-based gene predictions. Other parameters in the MAKER annotation pipeline were set as default. We discarded overlapping models as well as protein models with fewer than 25 translated amino acid residues from the final protein model. Orthologue-based gene annotation was conducted in eggNOG-mapper 2.1.7 [40]. Protein domain annotation was conducted with InterProScan 5.54–87.0. For consistency, we also applied this pipeline to re-annotate the genome of *B. bassiana* ARSEF2860, which was included as an external reference for phylogenomic analysis and for anchoring targeted comparative genomics.

### Phylogenomics

Twenty-four reference genomes representing Cordycipitaceae, plus that of *M. acridum* CQMa 102 representing Clavicipitaceae (outgroup), were selected for phylogenomic analyses. We first extracted benchmarking universal single-copy orthologues (BUSCOs) from the 25 genome assemblies using BUSCO 5.4.7 with the Kingdom Fungi Odb10 reference. The BUSCO genes found in ≥23 taxa were used for protein sequence alignment using MAFFT 7.490 [41]. The alignments were trimmed for informative sites using GBlocks 0.91 [42]. Based on these criteria, 240 BUSCO genes with informative sites were used for phylogenetic reconstruction.

The species tree inference was performed using two approaches: maximum likelihood (ML)-based inference from multi-locus concatenation and gene tree-species tree coalescence. For the first approach, we concatenated the trimmed alignments of the 240 BUSCO genes into a single alignment, which was used as input data for ML species tree construction performed in RAxML 8.2.12 [43] with JTTG as an amino acid substitution model and 1,000-replicate bootstrapping. For the second approach, we estimated an optimal amino acid substitution model for each BUSCO trimmed alignment with Prottest 3.4.2 [44]. This optimal model was used for a gene tree reconstruction using the ML approach with 100-replicate bootstrapping. Then, the set of 240 gene trees was used for coalescence-based species tree inference in Astral-III 5.7.3 [45]. The resulting species trees from the two approaches (ML concatenation and gene tree-species tree coalescence) were merged and compared to assess topological consistency.

### Detection of gene presence–absence patterns

Orthology assessment of 17 *Beauveria* genomes (15 genomes assembled *de novo*, *B. neobassiana* BCC 2660 and *B. bassiana* ARSEF2860) was performed using OrthoFinder 2.5.4 [46]. The gene copy numbers among gene families and strains were transformed to count data and analysed for gene family contraction/expansion across the 17 genomes using Count 9.1106 [47]. To infer gene gain and loss events along the phylogenomic tree, we ran Wagner parsimony analysis using orthogroups present in at least two genomes (Table S2) and the consensus species tree from the phylogenomic analysis above as the backbone tree. Additionally, strain-specific orthogroups were identified using an *in-house* script. In particular, we identified orthogroups uniquely found in high-virulence or low-virulence strains. Orthogroups that are present in at least two genomes of high-virulence strains but absent in all genomes of low-virulence strains were examined for putative functions through eggNOG annotation and signal peptides predicted by SignalP-6.0 [48].

### Genome scan of gene presence–absence associated with virulence

We analysed gene presence–absence across the 16 strains with known virulence to identify orthogroups whose distribution patterns correlated with virulence, using targeted and non-targeted approaches. For the non-targeted approach, we ran our comparative analyses in three layers. The first layer uses a metric called the ‘profile match score’ (PMS), which is an orthogroup-level score calculated by summing strain-wise binary scores across all genomes. Specifically, each genome was scored as 1 if the orthogroup was present (at least one copy) in a high-virulence strain or absent in a low-virulence strain, and 0 otherwise. This approach identifies orthogroups that tend to be consistently present in high-virulence strains and absent in low-virulence strains. Orthogroups with the top 5% of PMS (i.e. PMS ≥11 in our dataset) were considered ‘high profile match’ orthogroups (Table S3). The top 5% threshold represented the extreme upper tail of the empirical PMS distribution.

The second layer uses a metric called the ‘virulence pair score’ (VPS), which reflects intraspecific patterns of gene presence–absence correlated with virulence level within each species. For each *Beauveria* species that includes both high- and low-virulence strains (i.e. *B. bassiana*, *B. namnaoensis*, *B. neobassiana* and *B. thailandica*), a species-wise score of 1 was assigned if the orthogroup was present in all high-virulence strains and absent in all low-virulence strains. Therefore, the VPS can range from 0 (no such pattern in any species) to 4 (most consistent pattern across all four species). This comparison is performed to rule out effects of phylogenetic relatedness that could influence the gene presence–absence pattern.

We then selected only orthogroups that have PMS ≥11 and VPS ≥1 as putative virulence-associated orthogroups for further analyses in subsequent layers (Table S3). To assess whether these candidates exhibited enrichment in gene gain or loss events, we compared their presence–absence patterns to genome-wide expectations. Specifically, using the results from gene family evolution via Wagner parsimony, we computed odds ratios as the proportion of gain or loss events among putative virulence-associated orthogroups relative to the genome-wide orthogroups. Branches with odds ratios exceeding the mean plus 1.5 standard deviations across all branches were considered to show significant changes.

Finally, the third layer determined differentially expressed genes under insect-related conditions, as supporting evidence for virulence-associated genes. We conducted a literature search for transcriptomic studies in *B. bassiana*. Only one study, by Wang *et al*. [26], performed a standardized differential gene expression analysis with available protein IDs from *B. bassiana* ARSEF2860. Their study utilized insect cuticle extract as a stimulus to determine genes that were overexpressed in a high-virulence strain (GXsk1011) compared to a low-virulence strain (GXtr1009). Accordingly, we used the gene IDs from that study to compare with our putative virulence-associated orthogroups. Any candidate genes that had a corresponding gene identified by Wang *et al*. [26] were considered to have putative roles in virulence.

The putative virulence-associated orthogroups resulted from the three layers above were further examined for their functional role based on the eggNOG-mapper annotation and SignalP assignment as signal peptide. Enrichment analysis was performed through a hypergeometric test to assess whether any functional classes in eukaryotic clusters of orthogroups (KOG) or proteins with a signal peptide were significantly enriched among putative virulence-associated orthogroups. Briefly, the test determines whether orthogroups in each functional class were significantly overrepresented among putative virulence-associated orthogroups compared to all orthogroups in all classes. We used the function ‘phyper’ implemented in R 4.4.1 (RStudio 2024.04.02) for the hypergeometric test. The presence–absence patterns of putative virulence-associated orthogroups were displayed as a heatmap using pheatmap 1.0.12 [49].

Since the comparative analyses here primarily relied on gene presence–absence patterns across strains with different virulence levels, we tested whether virulence measures (mortality percentages and mycelial colonization percentages) were correlated with predicted gene counts (Fig. S1E, F). This was done to ensure that observed differences were not biassed by genome annotation quality or gene model quantity.

### Genome scan of positive selection

We tested for genes that were under positive diversifying selection. First, 5,366 single-copy orthogroups from all 16 *Beauveria* strains were retrieved from the OrthoFinder results. DNA coding sequences of each single-copy orthogroup were aligned at the codon level using PRANK v140603 [50]. From this step, 256 orthogroups were discarded due to incomplete sequences or poorly predicted genes (i.e. having at least one genome with a misaligned region more than 50% of the total alignment length), leaving 5,110 alignments for further analysis. Gene trees were reconstructed from these alignments using FastTree v2.1.11 [51]. We then detected positive selection specific to high-virulence strains with the adaptive branch-site random effect likelihood (aBSREL) method implemented in HyPhy package v2.5.36 [52,53], using the orthogroup gene trees from FastTree as inputs. Any orthogroups that contain at least one orthologue with a significant aBSREL test were compared to the differentially expressed genes from Wang *et al*. [26] and examined for functional roles (eggNOG-mapper) and putative signal peptide (SignalP). Finally, we reconstructed a heatmap using pheatmap 1.0.12 to visualize the patterns of genes under positive selection across the 16 *Beauveria* strains.

### Targeted CAZyme analyses

Besides the non-targeted analyses described above, we focused on carbohydrate-active enzymes (CAZymes) with specificity to chitin (glycoside hydrolase families GH18 and GH19; carbohydrate esterase family CE4; and auxiliary activity families AA10, A11 and A15). These groups are of interest as they were previously reported to affect insect pathogenicity and virulence of *Beauveria* species [8,54].

We identified genes encoding chitin-specific CAZymes in all analysed genomes by utilizing dbCAN 3.0.2 [55], based on three methods: DIAMOND [56], eCAMI [57] and HMMER [58]. Only protein sequences with concordant predictions from at least two methods were retained. We subset CAZymes with chitin specificity following Miyauchi et al. [59]. Our dataset has three chitin-specific CAZyme families: auxiliary activity family 11 (AA11), carbohydrate esterase family 4 (CE4) and glycoside hydrolase family 18 (GH18). Given that chitin-specific CAZyme orthogroups are generally conserved across strains, we did not use presence–absence patterns for virulence association but instead focused on testing whether they exhibit gene copy number variation or signatures of positive selection.

Gene count distributions for these chitin-specific CAZyme families were obtained and visualized as a heatmap constructed with pheatmap v1.0.12. Summation of gene counts for these chitin-specific CAZyme families was performed using an in-house script. We tested for statistical difference in chitin-specific CAZyme gene copy numbers between high- and low-virulence strains using a generalized linear model (GLM). Virulence level (high versus low) was included as a categorical predictor, and gene count was modelled as the dependent variable under a negative binomial distribution (Gene count ~ virulence level); test results were corrected for multiple testing by FDR method using multcomp v1.4–14 package [60] performed in R v4.4.1.

Subsequently, we focused on chitin-specific CAZymes annotated in ARSEF2860 and examined the corresponding orthogroups in the other 16 strains. We visualized gene copy number variation using a heatmap and tested for positive selection in high-virulence strains using the aBSREL method as described above. The transcriptomic dataset from Wang *et al*. [26] was also mapped to find correspondence with our CAZyme-coding genes.

### Targeted analysis for biosynthetic gene clusters

Another targeted genes of interest comprised those within secondary metabolite biosynthetic gene clusters (SMBGCs) encoding NRPS. Biosynthetic clusters in all 17 analysed *Beauveria* genomes were predicted using the web-based fungal antiSMASH v7.1 [61]. We selected only SMBGCs with blast hits to known clusters in the antiSMASH database. Based on presence–absence patterns across the 17 genomes, each known SMBGC was assigned for PMS and VPS. The distribution of these SMBGCs was visualized as a heatmap with pheatmap v1.0.12. To further investigate NRPS gene evolution, we retrieved NRPS clusters annotated by antiSMASH in *B. bassiana* ARSEF2860. The analysis pipeline including orthology assessment, sequence alignment, orthogroup gene count, aBSREL test and transcriptomic data mapping was performed as described for chitin-specific CAZymes.

## Results

### Virulence assay

**Fig. 1.**
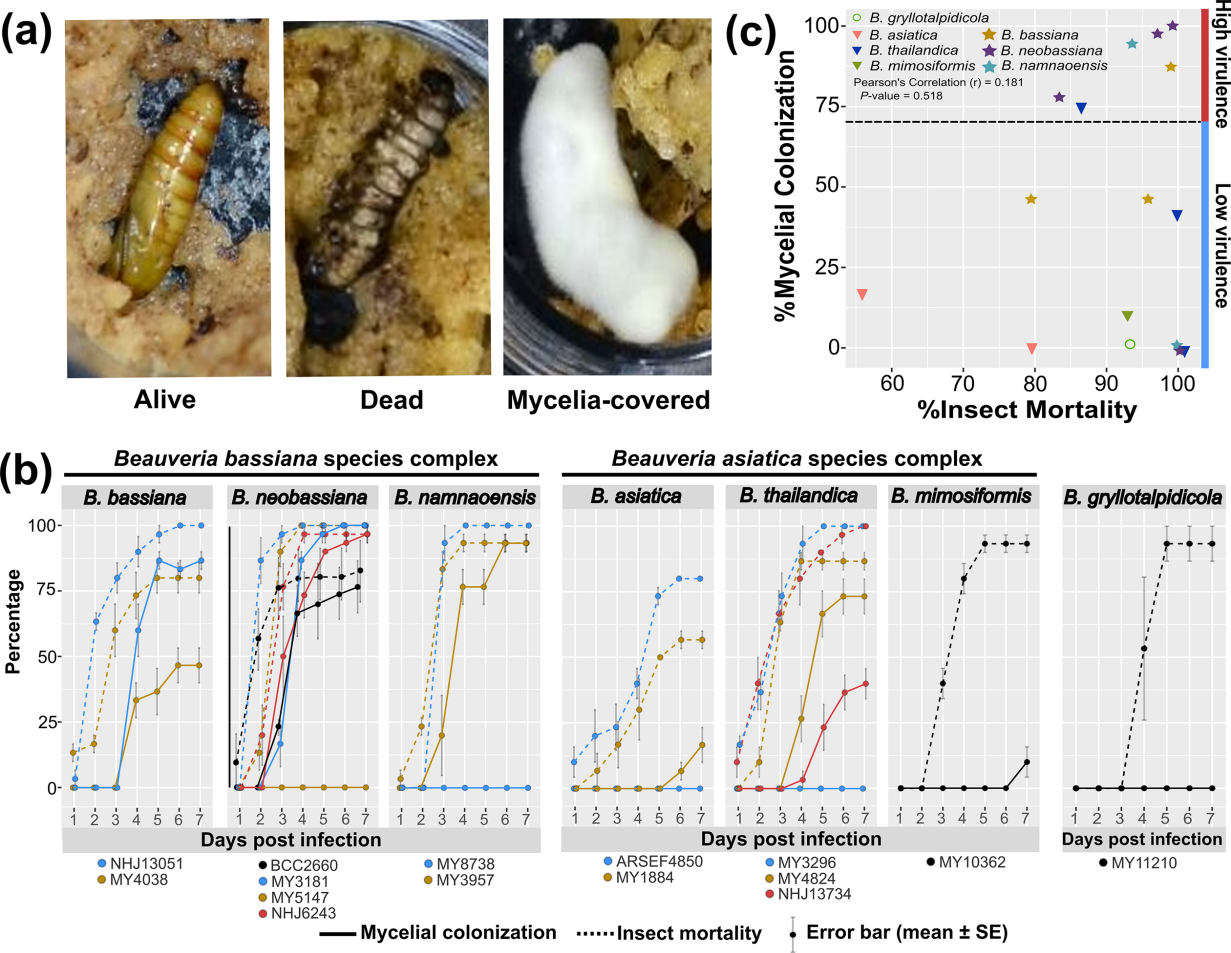
Virulence assay of *Beauveria* strains against beet armyworms (*Spodoptera exigua*). Fifteen *Beauveria* strains were tested for virulence against the beet armyworm. Thirty worms, divided into three replicates of ten individuals, were inoculated by each strain. Two values were measured in percentages: insect mortality (indicated by immobility of worms and changed body colour to dark brown/black) and mycelial colonization (indicated by mycelia covered throughout the worm body). (**a**) Photographs showing the symptom of infection compared to control; healthy larvae have bright yellowish-brown colour and develop into a pupae stage (left), while dead larvae are either necrosed with dark colour (middle) or covered by mycelia (right). (**b**) Intraspecific and interspecific variability of virulence based on time-course experiments. Line graphs represent time-course data from day 1 to day 7 after inoculation. (**c**) The scatterplot showing insect mortality (%) and mycelial colonization (%) after 7 days of inoculation. Strains from *B. bassiana* species complex are mostly more virulent than the ones from *B. asiatica* complex. Note that *B. neobassiana* BCC2660 is referenced as a high-virulence strain based on our previous study.

Our time-course experiments evaluating the virulence of *Beauveria* strains against beet armyworms (*S. exigua*) revealed notable variability in virulence at both intra- and interspecific levels (Fig. 1). The virulence was assessed using two proxies: (i) mortality, defined as the percentage of insects dead at 7 days post-infection, and (ii) mycelial colonization rate, defined as the proportion of cadavers exhibiting visible external mycelia. All tested strains, except one (*B. asiatica* MY1884), were able to inflict at least 80% mortality to *S. exigua* after 7 days of inoculation, while only six strains were able to develop the mycelial colonization to more than 70% of treated insects (Fig. 1). In most cases, we observed a lag period of 1–3 days before the mycelial colonization became apparent. Only for certain cases, the mycelial colonization reached the same level as the insect mortality. Three out of four strains of *B. neobassiana* (BCC2660, MY3181 and MY5147) and one each from *B. bassiana* (NHJ13051) and *B. namnaoensis* (MY3957) caused an equivalent mycelial colonization to the mortality. In other instances, no visible mycelial colonization was detected on host surfaces despite insect mortality rates exceeding 90% (*B. gryllotalpidicola* MY11210, *B. namnaoensis* MY8738, *B. neobassiana* MY5147 and *B. thailandica* MY3296). Overall, a correlation test between these two proxies at 7 day post-infection indicated no significant linear relationship (Pearson’s correlation test: *n*=16, *r*=0.181, *P*-value=0.518, Fig. 1). Considering the lack of correlation between both parameters, the mycelial colonization rate is preferred since the mycelial colonization provides a more stringent and ecologically relevant measure of fungal fitness as it reflects both host-killing ability and the capacity for environmental persistence and potential secondary infection. This approach is also supported by recent studies selecting biocontrol strains consistently achieving the highest mortality and mycelial colonization concordantly [62,63].

Strains with <70% mycelial colonization were thus considered lowly virulent, whereas those with ≥70% were considered highly virulent. Based on these criteria, six strains comprising five from *B. bassiana* species complex (*B. bassiana* MY4038; *B. namnaoensis* MY3957; and *B. neobassiana* BCC2660, MY3181 and NHJ6243) and one from *B. thailandica* (MY4284) were considered highly virulent, while ten other strains, mostly from *B. asiatica* complex, were considered lowly virulent (Fig. 1). Besides these two species complexes, *B. gryllotalpidicola*, represented by a unique strain in this study, was classified as lowly virulent with mycelial colonization (%) of zero.

### Genome statistics

The features of 15 *Beauveria* draft genomes generated in this study are summarized in Table S1 and Text S1 (Files S1 and S2). Despite various N50 values (39.6–196.7 kb) and coverage depth (13×–126×) across the assemblies, high BUSCO completeness percentages (98.2–99.6%), indicate excellent coverage of genic regions in these assemblies, which would provide robustness on subsequent comparative genomic analyses.

### Phylogenomics

The species tree reconstructed from concatenation of 240 BUSCO genes and that of the gene tree-reconciled coalescence approach yield identical tree topologies (Fig. S2). Consistent with the species complexes defined by Kobmoo [1], two major clades corresponding to *B. asiatica sensu lato* (comprising *B. asiatica, B. thailandica* and *B. mimosiformis*) and *B. bassiana sensu lato* (comprising *B. bassiana*, *B. neobassiana* and *B. namnaoensis*) were identified. Additionally, *B. brongniartii* RCEF3172 clustered with the *B. asiatica* species complex clade, whereas *B. pseudobassiana* RGM2184 and *B. gryllotalpidicola* MY11210 represented more early-diverging lineages in the genus *Beauveria*. Our analysis further revealed that *B. bassiana* ARSEF2860, commonly utilized for the genome reference [19], clustered with *B. neobassiana*, but not with *B. bassiana sensu stricto* (Fig. S2), suggesting the affiliation of this strain to the former and not the latter.

### Identification of virulence-associated orthogroups from gene presence–absence pattern

**Fig. 2.**
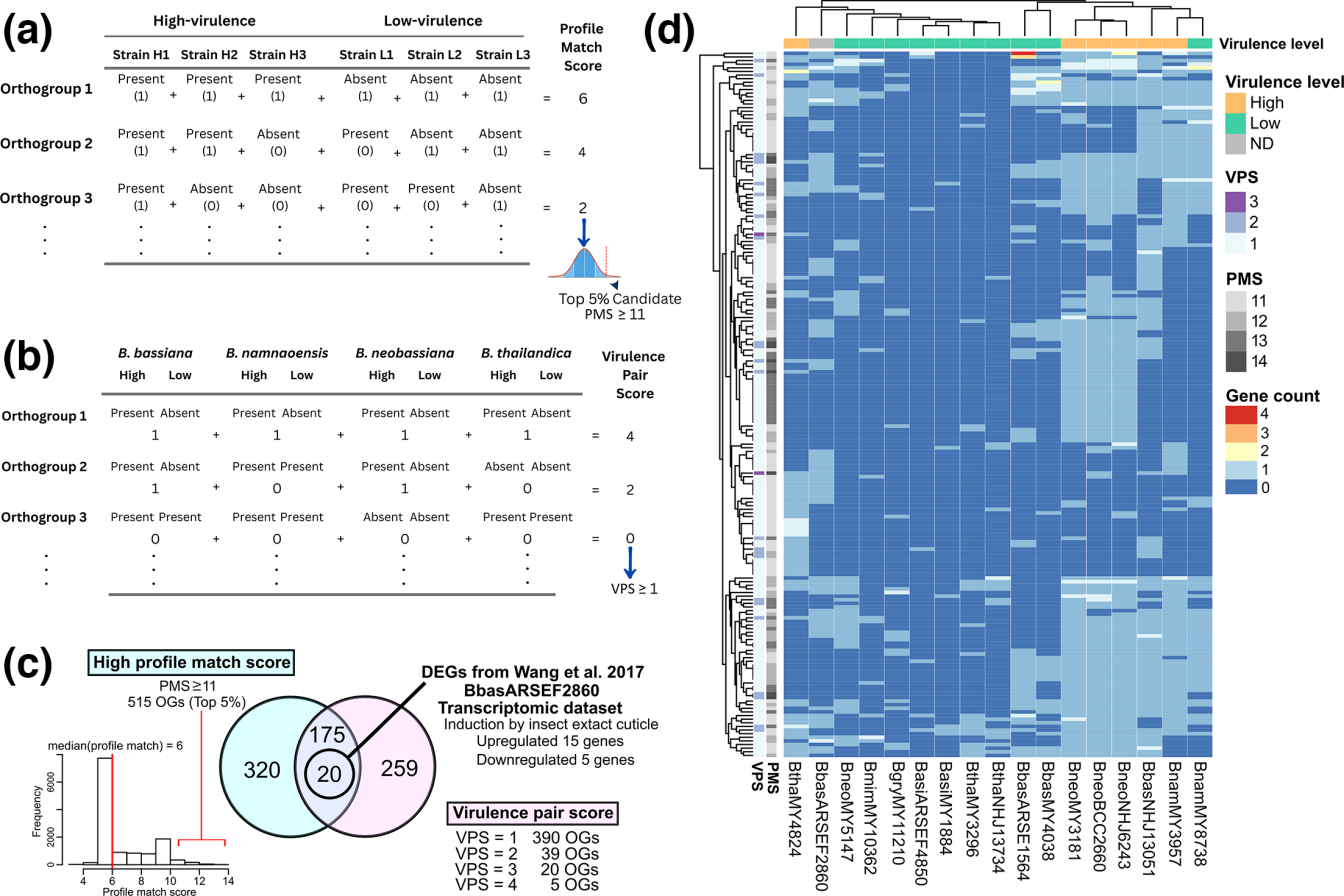
Presence/absence patterns of putative virulence-associated orthogroups among 17 *Beauveria* genomes. (**a, b**) Schematic diagrams showing the calculation of profile match score, abbreviated as PMS (**a**) and virulence pair score, abbreviated as VPS (**b**). (**c**) Venn diagram showing a number of putative virulence-associated orthogroups under three criteria: PMS, VPS and differentially expressed genes from transcriptomics. (**d**) Heatmap showing gene count distribution of 195 putative virulence-associated orthogroups that have high PMS (≥11) and VPS (≥1). Row and column clustering is based on a similarity of presence/absence patterns determined by Euclidean distances. Only orthogroups present in at least 2 genomes, 10,652 orthogroups in total, were used as an input for the association analyses.

We first conducted a heuristic non-targeted comparative genomic analysis across 17 *Beauveria* genomes to identify candidate genes potentially involved in virulence. Out of 10,652 orthogroups found in at least 2 genomes (Table S2), we identified 515 orthogroups with PMS ≥11 and 454 orthogroups with VPS ≥1 (Table S3–S4). Among these, 195 orthogroups satisfied both criteria simultaneously (Fig. 2, Table S5) and were then considered putative virulence-associated orthogroups. Further integration with gene expression data [26] revealed that 20 of these orthogroups were differentially expressed (15 upregulated, 5 downregulated) between the high-virulence strain GXsk1011 and the low-virulence strain GXtr1009 of *B. bassiana* upon induction with insect cuticle extract (Table 1). Notably, 10 of these 20 putative virulence-associated orthogroups lacked functional annotations from eggNOG-mapper or InterProScan. Among the functionally annotated orthogroups upregulated in GXsk1011 (|log2 fold change|≥1) were genes encoding F-box and WD repeat-containing protein (*BBA_01737* in *B. bassiana* ARSEF2860), a major facilitator superfamily (MFS) transporter (*BBA_00223*), a glutamine synthetase family protein (*BBA_04942*) and a sterol desaturase family protein (*BBA_05271*).

**Table 1.** List of putative virulence-associated orthogroups from the analyses across 16 *Beauveria* genomes. Only orthogroups having respective orthologues in *B. bassiana* ARSEF2860, profile match score (PMS) ≥11 (Top 5%) and virulence pair score (VPS) ≥1 are included in the table. The full list of putative virulence-associated orthogroups from PMS and VPS can be found in Table S3–S5

Orthogroup	Reference gene from *B. bassiana* ARSEF2860	Consensus annotation from eggNOG-mapper, blastp and InterProScan	PMS	VPS	Log2FC from Wang *et al*. [26] study*
OG0010325	BBA_02890	Hypothetical protein	11	1	−11.99
OG0009367	BBA_08375	Phosphotransferase enzyme family	11	1	−4.54
OG0009256	BBA_05667	Hypothetical protein	13	1	−2.39
OG0008695	BBA_09405	Ceramidase	12	1	−1.44
OG0009289	BBA_09797	GAL4-like Zn(II)2Cys6 (or C6 zinc) binuclear cluster DNA-binding domain	12	1	−1.19
OG0009237	BBA_06796	WD40 repeat domain protein	11	1	1.06
OG0009590	BBA_04176	MFS	13	1	1.48
OG0009342	BBA_05167	CaaX prenyl protease	13	2	1.61
OG0009349	BBA_07390	Hypothetical protein	14	2	1.76
OG0009581	BBA_04484	Hypothetical protein	12	1	2.05
OG0008971	BBA_09596	Succinate dehydrogenase cytochrome	11	1	2.09
OG0009451	BBA_00368	F-box domain protein	12	1	2.24
OG0009588	BBA_08713	Hypothetical protein	12	1	2.36
OG0009700	BBA_01737	F-box and WD repeat-containing protein	11	1	3.95
OG0009338	BBA_00223	MFS	12	1	4.30
OG0009072	BBA_03706	Hypothetical protein	12	1	9.00
OG0009178	BBA_05089	Hypothetical protein	12	1	9.96
OG0008632	BBA_04942	Belongs to the glutamine synthetase family	11	1	10.07
OG0009121	BBA_05271	Belongs to the sterol desaturase family	11	1	11.05
OG0008858	BBA_09865	GTPase	11	1	12.33

*Log2FC values are indicated for only genes that are differentially expressed in the high-virulence strain GXsk1011 compared to the low-virulence strain GXtr1009.

Among the 195 orthogroups meeting both selection criteria (PMS ≥11 PMS and VPS ≥1), only 28 orthogroups had functional assignments provided by eggNOG-mapper (Table S5), and none of any functional classes were significantly enriched (Table S6). Some of these annotated genes are known to be associated with fungal pathogenicity and virulence [8,10]. Notable examples include genes encoding a Cytochrome P450 (*BBA_02747* in *B. bassiana* ARSEF2860), an MFS transporter (*BBA_00068*, *BBA_00223* and *BBA_04176* in *B. bassiana* ARSEF2860) and proteases and peptidases (*BBA_00069*, *BBA_05167* and *BBA_08694* in *B. bassiana* ARSEF2860).

### High virulence-specific orthogroups

In addition to the heuristic scoring of gene presence–absence above, we also examined orthogroups that were present only in high-virulence strains but absent in all low-virulence strains (Fig. S3, Table S4). Overall, 7,474 orthogroups were shared by all strains. A total of 454 orthogroups were unique to high-virulence strains; the majority (390) were strain-specific, while only a few were shared by multiple high-virulence strains – 39, 20 and 5 orthogroups were shared by 2, 3 and 4 high-virulence strains, respectively (Table S4). Results from eggNOG-mapper revealed only 63 of the 454 orthogroups had assigned functions, and none of these functional classes were significantly enriched (Table S6). Among the orthogroups shared by four high-virulence strains, only one orthogroup had a known function (predicted as an HNH endonuclease; *BCC2660_009367*). Three of the 20 orthogroups present only in three high-virulence strains had functional annotations: OG0010695 (*BCC2660*_*009917*) encoding a zinc finger protein, OG0010715 (*BCC2660_009221*) encoding a protein with FUN14 domain and OG0010925 (*BbasNHJ13051_003577*) encoding a FAD-binding protein. Nine out of 39 orthogroups shared by 2 high-virulence strains had annotated functions (Table S3), including OG0010682 (*BCC2660_002141*) predicted as an ABC transporter and OG0011321 (*BCC2660_008104*) predicted as a peptidase S10 family (Table S4).

### Gene gain and loss with respect to virulence

**Fig. 3.**
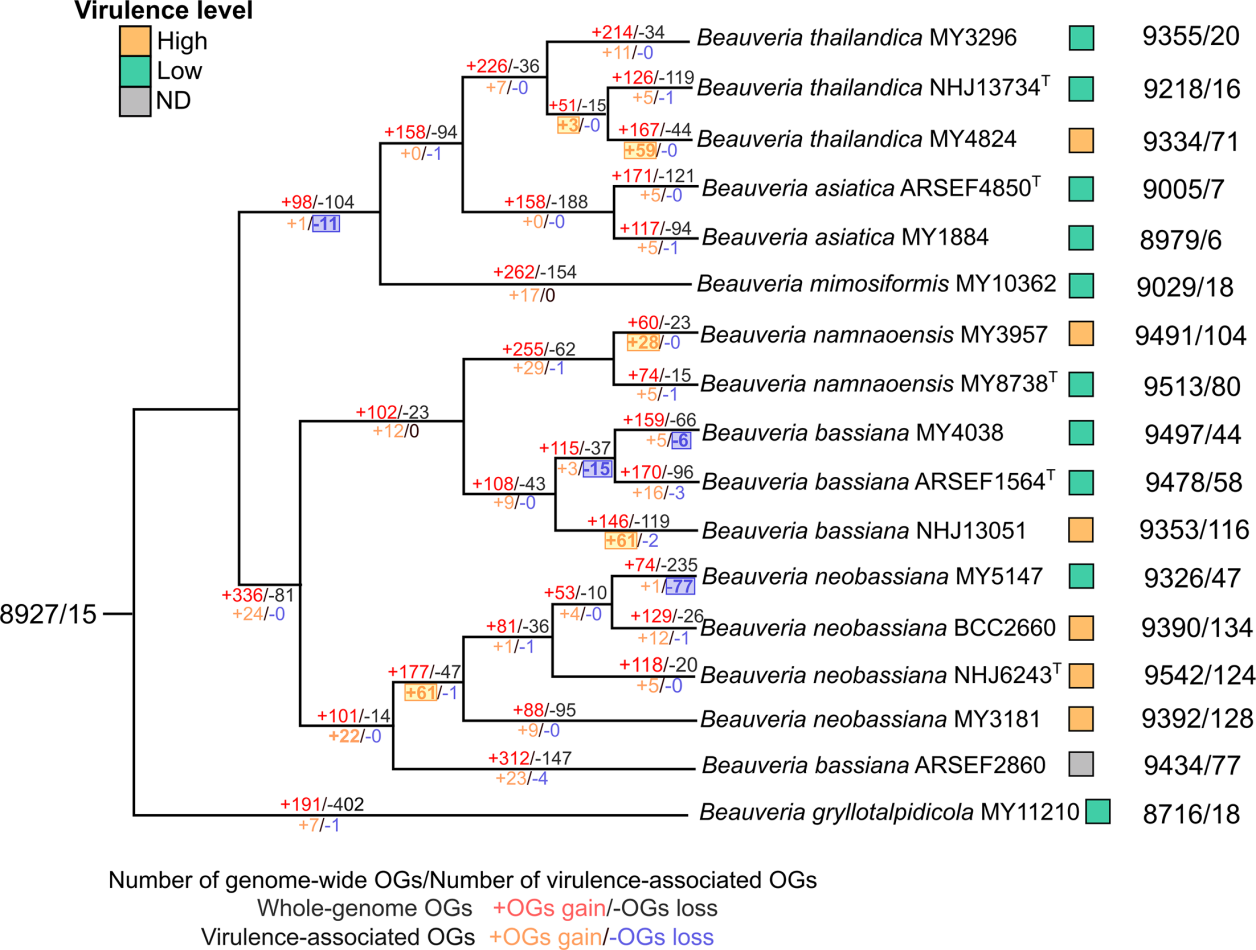
Gene gain/loss events inferred across 17 *Beauveria* genomes. A species tree reconstructed from phylogenomic analyses was used as a guide tree for estimating evolutionary histories of gene gain/loss through Wagner parsimony performed in count 9.1106. Numbers at the branches indicate amounts of gained/lost orthogroups at the closest node for the whole-genome dataset (above the branches, in red/black) and for putative virulence-associated orthogroups (below the branches, in orange/blue texts), respectively. Numbers on the right side of the tree indicate numbers of genes found in each genome for whole-genome/virulence-associated orthogroup sets. At the root node, the numbers of estimated orthogroups of the most recent common ancestor of the strains are indicated. Bold texts with highlighted boxes indicate nodes with significant events of gene gain/loss of virulence-associated orthogroups that exceed an average odds ratio compared to the whole-genome dataset. Colour keys indicate virulence levels for each *Beauveria* strain.

At the genome-wide scale, reconciliation of gene gain/loss events (Fig. 3) showed the highest orthogroup gains (336) at the common ancestor node of *B. bassiana*, *B. neobassiana* and *B. namnaoensis*, followed by the node diverging to *B. bassiana* ARSEF2860 (312 gains). Conversely, the terminal branches of *B. gryllotalpidicola* MY11210 and *B. neobassiana* MY5147 exhibited the highest orthogroup losses, with 402 and 235 losses, respectively. We then calculated the odds ratios for gene gain–loss events for the 195 candidate orthogroups relative to genome-wide expectations (Fig. 3). Among the terminal branches, the strongest enrichment of gene gains in putative virulence-associated orthogroups was observed for *B. bassiana* NHJ13051 (+61, odds ratio: 27.4), *B. namnaoensis* MY3957 (+28, odds ratio: 30.7) and *B. thailandica* MY4824 (+59, odds ratio: 23.2). Notably, these terminal branches represent transitions from low to high virulence with regard to their closely related sister strains, all considered low virulence (Fig. 3). In addition, two internal nodes displayed gene gains with significant odds ratios, suggesting significant events associated with increased virulence: the node ancestral to *B. neobassiana* (+61, odds ratio 22.7) and the node of the common ancestor between *B. neobassiana* and *B. bassiana* ARSEF2860 (+22, odds ratio 14.3). *B. neobassiana* is the most virulent species according to our virulence assays, suggesting that gene gain events may have contributed to increased virulence.

Conversely, several nodes, leading to typically low-virulence strains, were characterized by gene losses with significant odds ratios. For example, two terminal nodes exhibited significant gene losses above the average odds ratio (2.5). These are nodes for *B. neobassiana* MY5147 (−77, odds ratio 21.5) and *B. bassiana* MY4038 (−6, odds ratio 6.0), two strains that were classified as lowly virulent. Two other internal nodes with gene losses exceeding the average odds ratio are the common ancestor node between MY4038 and ARSEF1564 (−15, odds ratio 26.7), the two low-virulence strains of *B. bassiana* and the ancestral node between *B. asiatica*, *B. mimosiformis* and *B. thailandica* (−11, odds ratio 7.0); these taxa were mostly classified as lowly virulent (Fig. 3).

### Identification of positively selected genes

**Fig. 4.**
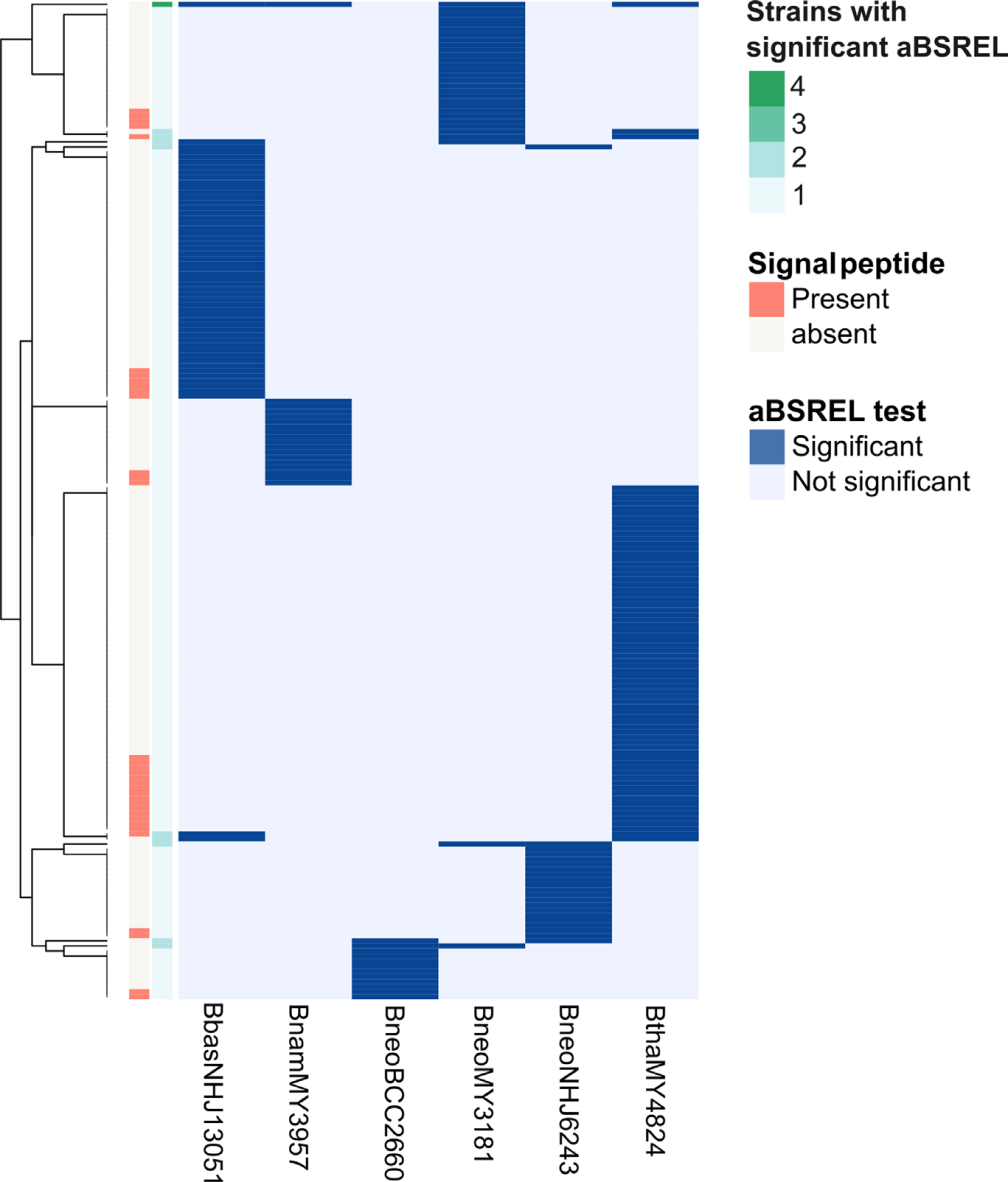
Heatmap showing orthogroups with significant positive selection detected in high-virulence strains following aBSREL test. A total of 5,110 single-copy orthogroups among 16 *Beauveria* strains with virulence data were subjected to aBSREL test (aBSREL test for episodic diversification) specifying the branches leading to the six high-virulence strains. Only 196 orthogroups have genes in at least 1 high-virulence strain with a significant aBSREL test. Each row indicates an orthogroup; each column indicates a high-virulence *Beauveria* strain. Cells with dark blue colour show significance from the aBSREL test. Colour codes on the left side indicate the number of high-virulence strains having aBSREL-positive genes (green shade), or the presence/absence of signal peptide for each orthogroup (orange shade). Euclidean distance is used for row clustering in the heatmap.

In addition to gene presence–absence patterns, we examined whether orthogroups experienced positive diversifying selection specific to high-virulence strains. From 5,110 single-copy orthogroups, we found 196 orthogroups with at least 1 orthologue significantly under positive selection (aBSREL test) in high-virulence *Beauveria* strains. Among these, one orthogroup exhibited significant selection signals in orthologues from four high-virulence strains, and nine orthogroups demonstrated significant signals of selection in two high-virulence strains. These are genes for transporters, receptor binding, peptidases and proteins without known function (Table 2 and S7, Fig. 4). Functional enrichment analysis of the 196 orthogroups with positive selection revealed that class S (function unknown) is the only KOG class that is enriched (62 out of 1,315 orthogroups assigned in class S, odds ratio=1.35, *P*-value=0.024, Table S8). However, orthogroups with signal peptides are significantly enriched among orthogroups subjected to positive selection (34 orthogroups with positive selection from the total 466 orthogroups with detected signal peptides, odds ratio=2.18, *P*-value=6.57×10^−5^). Mapped to the dataset of Wang *et al*. [26], 38 out of 196 orthogroups correspond to genes up-regulated in the high-virulence strain GXsk1011 of *B. bassiana*. Twelve of 38 orthogroups were predicted to encode secreted proteins following the presence of signal peptides detected with SignalP (Table S7). The positive selection detected in these orthogroups was strain-specific (Fig. 4). *B. thailandica* MY4824 and *B. bassiana* NHJ13051 are the two strains with the highest number of positively selected genes (73 and 54 genes, respectively). Some orthogroups under positive selection in specific high-virulence strains were annotated with functions potentially related to virulence. For example, OG0003769, identified under positive selection in NHJ13051, encodes a putative secreted chitinase (GH18 family), while OG0001005, annotated as a serine decarboxypeptidase belonging to S10 peptidase family, was found under positive selection only in MY4824. This corresponds to the phylogenomic result that each of these two strains is found as high virulence among sister strains in the species that are low virulence (Fig. S2).

**Table 2.** List of single-copy orthogroups with positive selection based on aBSREL tests. Only orthogroups having significant aBSREL tests in at least two high-virulence strains are included in the table. The full list of single-copy orthogroup with significant aBSREL tests can be found in Table S7

Orthogroup	Reference gene from *B. neobassiana* BCC2660	Consensus annotation from eggNOG-mapper, blastp and InterProScan	KOG class*	SignalP	A no. of strains with significant aBSREL tests	Log2FC from Wang *et al*. [26] study†
OG0002408	BCC2660_009845	Envelope protein	VW	OTHER	4	1.585
OG0008189	BCC2660_009875	Hypothetical protein	–	SP	2	2.562
OG0007646	BCC2660_001710	Endo beta glucanase	–	OTHER	2	2.507
OG0003765	BCC2660_008013	Complex I intermediate-associated protein 30	S	OTHER	2	1.601
OG0007691	BCC2660_009784	Conserved expressed protein	S	OTHER	2	1.136
OG0005745	BCC2660_005891	Peptidase family M3	O	OTHER	2	−1.516
OG0002475	BCC2660_006957	TAP-like protein	S	SP	2	na
OG0008094	BCC2660_006102	Hypothetical protein	–	SP	2	na
OG0003365	BCC2660_001702	Carbamoyltransferase	–	OTHER	2	na
OG0006460	BCC2660_003940	X-Pro dipeptidyl-peptidase	S	OTHER	2	na

*Classification of eukaryotic clusters of orthogroups (KOG): class O, posttranslational modification, protein turnover and chaperones; class V, defence mechanisms; class W, extracellular structures; class S, function unknown.

†Log2FC values are indicated for only genes that are differentially expressed in the high-virulence strain GXsk1011 compared to the low-virulence strain GXtr1009. na, not differentially expressed in Wang *et al*. [26] dataset.

### SMBGC analyses

**Fig. 5.**
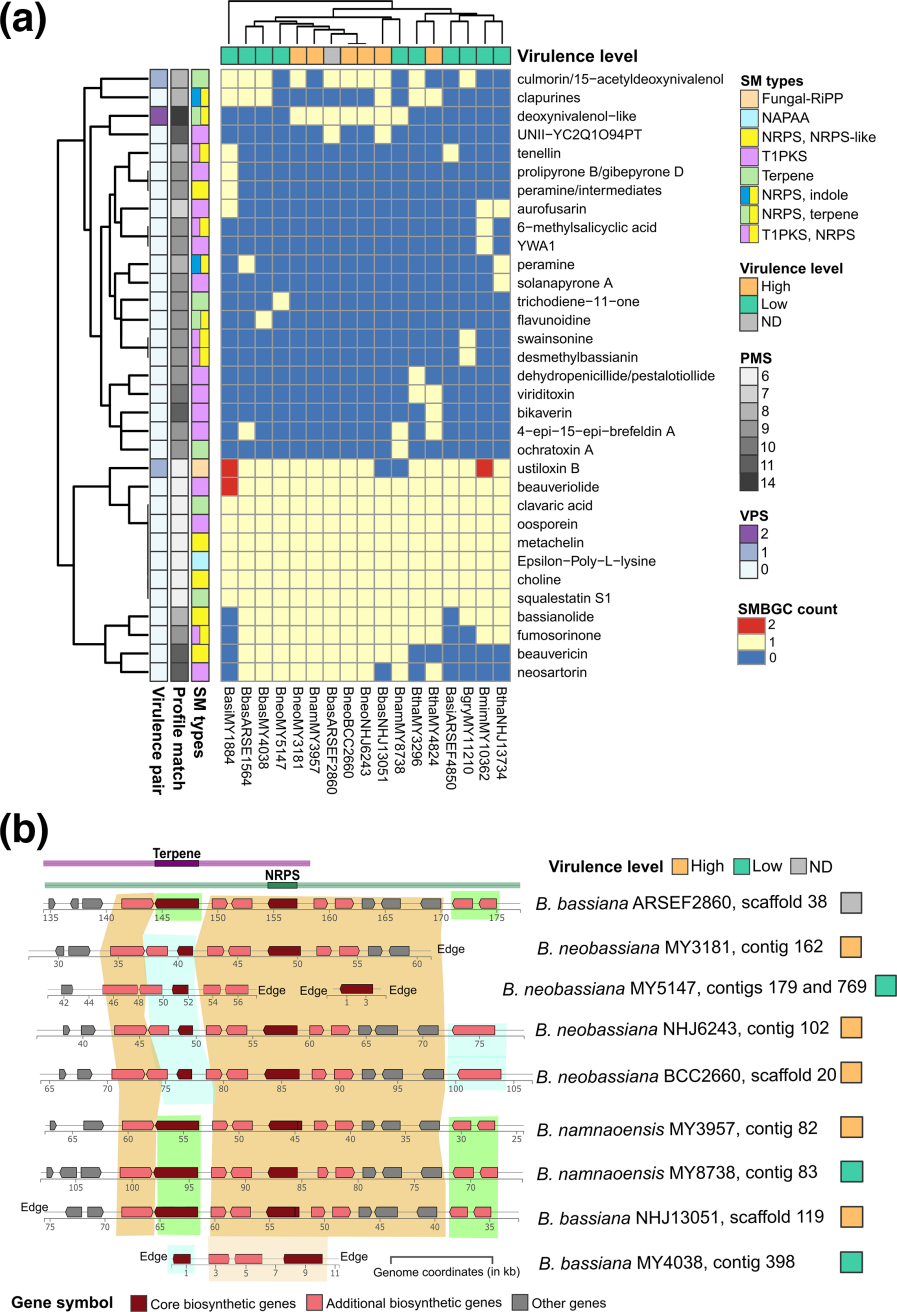
Comparative SMBGC analyses. (**a**) Heatmap showing the distribution of SMBGCs (annotated by antiSMASH) with known synthesized secondary metabolites among 17 *Beauveria* genomes. Row and column clustering is based on similarity of presence/absence patterns determined by Euclidean distances. Only an SMBGC for synthesizing a deoxynivalenol-like compound has high PMS (14) and VPS (2), implying that this SMBGC may affect virulence for insect pathogenesis. (**b**) Synteny analysis of secondary metabolite gene cluster (SMBGC) for deoxynivalenol-like compound. This cluster contains two core biosynthetic genes – one for NRPS and the other for the terpene biosynthetic gene. Different shading colours (orange, green or blue) indicate syntenic genes across nine genomes in the analysis. Note that there are two orthogroups of terpene biosynthesis gene: OG0009730 found in *B. neobassiana* and *B. bassiana* MY4038, and OG0008483 found in the other strains. Note that *B. bassiana* ARSEF1564 lacks this gene cluster due to the absence of OG0000659 and OG0009730 in its genome.

Annotation of SMBGCs in the 17 *Beauveria* genomes using antiSMASH identified 55.6±2.9 SMBGCs on average for each genome. Most of them were biosynthetic gene clusters for NRPS, averaging 21.4 clusters per genome (Table S9). Approximately 13.8 (24.8%) of the annotated SMBGCs matched known secondary metabolites. Based on heuristic analyses from gene presence–absence conducted previously, focusing on annotated SMBGCs, we found only one SMBGC that met the two criteria, e.g. high PMS (14) and high VPS (2). This cluster mostly matched a known gene cluster involved in the synthesis of a deoxynivalenol-like compound and was present in all high virulence *Beauveria* strains except *B. thailandica* MY4824 (Fig. 5a).

Synteny analysis of this SMBGC (Fig. 5b) revealed two types of biosynthetic core genes (Tables S2, S10): one NRPS core gene (OG0000659) or two terpene synthase core genes (OG0008483 and OG0009730). Orthogroup OG0000659 was detected in *B. namnaoensis*, *B. neobassiana* and most *B. bassiana* strains (except ARSEF1564). The orthogroup OG0009730 was found specifically in *B. neobassiana* and most *B. bassiana* strains (except ARSEF1564), while OG0008483 was broadly distributed among all strains of *B. namnaoensis*, *B. neobassiana* and *B. bassiana* and also present in *B. asiatica* MY1884. Two distinct combinations of these core genes define the SMBGC for the deoxynivalenol-like compound: OG0000659 with OG0008483 (in both *B. namnaoensis* strains and the high-virulence strain *B. bassiana* NHJ13051) or OG0000659 with OG0009730 (in all examined *B. neobassiana* strains and the high-virulence *B. bassiana* MY4038). However, in *B. bassiana* MY4038 and *B. neobassiana* MY5147, this SMBGC was not annotated as a complete deoxynivalenol-like cluster due to contig breaks resulting in incomplete synteny.

We then focused on core biosynthetic genes encoding NRPS, given their prevalence in *Beauveria* genomes and their established roles in insect pathogenicity and virulence [8,54]. Specifically, five NRPS clusters annotated in the reference *B. bassiana* ARSEF2860 putatively synthesize known secondary metabolites: *BBA_02630* for bassianolide, *BBA_06997* for a metachelin-like compound, *BBA_07339* for fumosorinone, *BBA_08222* for beauveriolide and *BBA_09727* for beauvericin [54]. We retrieved the core NRPS biosynthetic genes in *B. bassiana* ARSEF2860 along with respective orthologues in the other 16 genomes to conduct targeted tests for positive selection (aBSREL) and comparative analyses (Fig. S4). Our analyses revealed that most of the NRPS core genes with known compounds annotated in *B. bassiana* ARSEF2860 were conserved in all other *Beauveria* strains (Fig. S4A, Table S2). There were however exceptions: *BBA_10105* (OG0009493, unique to *B. bassiana* ARSEF2860 and ARSEF1564), *BBA_07548* (OG0009057, absent from *B. asiatica*, *B. thailandica* and *B. gryllotalpidicola*) and *BBA_04827* (OG0008298, absent from *B. asiatica* and *B. thailandica*). Four orthogroups exhibited significant positive selection in at least one high-virulence strain (Fig. S4A, Table S10): OG0000275 (orthologue: *BCC2660_009317*), OG0008298 (orthologues: *BCC2660_009456*, *BneoNHJ6243_001155*), OG0000867 (orthologues: *BCC2660_006899*, *BneoNHJ6243_003417*) and OG0001562 (orthologue: *BnamMY3957_005230*, core biosynthetic gene for fumosorinone). The positively selected NRPS core genes are detected in the high-virulence strains of *B. neobassiana* (three orthologues), followed by *B. namnaoensis* (one orthologue).

### CAZyme analyses

We focused on three CAZyme families that have substrate specificity for chitin: GH18, CE4 and AA11. The distribution of CAZyme count showed that the family GH18 has the highest abundance with an average of 19.4±1.7 genes per genome (Fig. S5A), followed by AA11 (average gene count 2.6±0.6) and CE4 (average gene count 2.1±0.9). GLM with gene count data (negative binomial distribution) found no statistical difference in gene abundance between genomes from low- and high-virulence strains (Z-score for virulence level=−0.237, *P*-value=0.813, Fig. S5B).

Subsequently, we examined all chitin-specific CAZyme orthogroups mapped to *B. bassiana* ARSEF2860. We found that most of the chitin-specific CAZyme orthogroups were conserved across all analysed genomes (Fig. S4B), except for four orthogroups: OG0004797 (*BBA_09259*), OG0007807 (*BBA_08395*), OG0008694 (*BBA_09406*) and OG0008916 (BBA_09585). The aBSREL tests of positive selection on orthogroups for chitin-specific CAZymes revealed two orthogroups with at least one orthologue under positive selection in high-virulence strains (Fig. S4B, Table S10): OG0003769 (*BbasNHJ13051_003448*, *BnamMY3957_001824*) and OG0008694 (*BbasNHJ13051_008922* and *BthaMY4824_008408*).

## Discussion

### Species-level patterns in virulence

Although *B. bassiana* has long been recognized as an effective biological control agent [3], limited consideration of species-level diversity within the genus has constrained our understanding of the factors underlying insect pathogenicity and virulence at the infra-generic level. With recent genome-scale taxonomic resolution of *B. bassiana* and *B. asiatica* species complexes [1], this study is among the first to integrate both pathological and comparative genomic analyses at a multispecies level. Our data showed substantial variability in *Beauveria* virulence toward *S. exigua* at both intra- and interspecific levels, with *B. bassiana* complex consistently exhibiting higher virulence compared to *B. asiatica* complex and *B. neobassiana* emerging as the most virulent species. Similar intra- and interspecific variability in virulence was also reported in Wang *et al*. [5], who found that species within *B. bassiana* complex exhibited generally higher virulence than those belonging to *B. scarabaeidicola* complex.

One significant finding from our study concerns how fungal virulence is measured. Although many publications do not clearly define their criteria for insect mortality [5,20, 22, 64,67], insect immobility is the most used practical proxy with optional observation of mycelial colonization. However, our study demonstrated that mortality does not always result in successful colonization, which we defined as the visible coverage of mycelia across the insect body (Fig. 1). While host mortality represents the classical definition of virulence, our insect mortality data showed that almost all the strains had >70% host mortality, limiting their ability to discriminate among pathogenic profiles. This discrepancy highlights the distinction between virulence and fitness. Traditionally, insect pathologists considered LD_50_ (a lethal dose causing 50% of insect death) or LT50 (a median time causing 50% of insect death) as proxies for virulence [68]. However, recent studies provided more ecological perspectives on pathogenic success by considering other traits related to fungal fitness such as sporulation and transmission rate [28,69]. Therefore, we considered mycelial colonization as an indicator of fungal fitness and successful completion of the infection cycle that requires insect mortality as a prerequisite. Future research on fungal–insect interactions should clearly define what is meant by ‘virulence’ and describe how it is measured. A more holistic approach may be necessary – one that includes multiple proxies such as insect mortality, colour change on host body and alteration of host developmental stages [23].

### Virulence candidates identified by gene presence–absence

Our comparative genomic analyses, based on gene presence–absence patterns, identified multiple classes of genes potentially contributing to virulence. Among these, over 70% have unknown functions, highlighting a trend in pathogen genomes in which a substantial fraction of virulence-associated genes lack known homologs or recognizable domains [70,72]. This pattern has also been consistently reported in genomic and transcriptomic studies of entomopathogenic fungi, including *Beauveria* and *Metarhizium*, where many candidate virulence genes appear lineage-specific or rapidly evolving and thus remain unannotated [9,19, 73, 74].

Despite the predominance of unannotated genes, our analyses identified several orthogroups with known or putative roles in virulence/pathogenicity (Table 1). A subset of these were previously shown to be differentially expressed between high and low-virulence strains in *B. bassiana* in response to insect cuticle exposure [9], making them particularly compelling. For example, OG0009289 (*BBA_09797*) encodes a zinc finger (Zn2Cys6) transcription factor. A recent study showed that some members in this Zn2Cys family (named *Rip1–Rip3*) are involved in *B. bassiana* virulence by affecting conidial viability, cuticle penetration and hyphal invasion [75]. Two other orthogroups, OG0009590 (*BBA_04176*) and OG0009338 (*BBA_00223*), encode MFS transporters. These transporters are involved in fungal morphology, stress response and pathogenicity in several plant pathogens including *Colletotrichum* spp. and *Botrytis cinerea* [76,78]. While MFS transporters in animal pathogens are primarily studied for roles in multidrug resistance [79,81], some have also been linked to virulence and biofilm formation in *Candida albicans* [82]. Additional candidates include genes involved in membrane lipid metabolism, such as OG0008695 (ceramidase) and OG0009121 (sterol desaturase), which may influence virulence by altering membrane properties and signalling pathways [83].

Beyond the differentially expressed genes reported by Wang *et al*. [26], we also identified several candidate orthogroups with known functions involved in fungal virulence (Table S3). For instance, OG0009225 (*BBA_00069 m.01*) encodes a metalloprotease – an enzyme class commonly involved in immune evasion, cell wall remodelling and host tissue degradation [84,86]. Furthermore, we identified several orthogroups specific to high-virulence strains that are functionally associated with pathogenicity in other systems (Table S4). An example includes a dipeptidyl peptidase; a member of this peptidase family plays a role in the pathogenesis of *Microsporum canis* by cleaving immune peptides and degrading host surface keratin [87]. However, the direct virulence role of this gene in *Beauveria* remains experimentally unverified. Other high-virulence-specific genes encode hydrolases, transporters and transcription factors – functional classes frequently implicated in fungal pathogenicity [8,81, 88].

### Evidence of positive selection

In addition to gene presence–absence patterns, we examined fast-evolving genes (determined by positive diversifying selection) as another means for detecting candidate virulence-associated genes [89]. Although we did not find any KOG classes with known function enriched among positively selected orthogroups, genes having signal peptides were significantly overrepresented (Tables S4, S5). This suggests strong selective pressure on secreted effectors for host–pathogen interactions, a common pattern for fungal pathogens [90,91]. Previous studies revealed several types of secreted proteins that play roles in *Beauveria* virulence against insect hosts such as hydrophobins, adhesin-like proteins, peptidases and chitinases [8,10]. Some of these genes were also found under positive selection in high-virulence strains of *Beauveria* in our study (Table S5). Most of the virulence-associated secreted proteins in *Beauveria* are involved in cuticle adhesion and cuticle penetration, while others play a role in degrading host tissues and counteracting host immunity [8]. Our results will serve as a foundation to investigate the role of these fast-evolving secreted proteins in insect pathogenesis through future genomic, secretomic and molecular genetic studies.

Although none of the orthogroups under positive selection in our targeted analyses were differentially expressed in the Wang *et al*. [26] dataset, some notable patterns emerged. First, *B. neobassiana* showed the highest prevalence of positively selected NRPS core genes among high-virulence strains, while no positively selected chitinase genes were detected in this clade. This, together with the emergence of a novel SMBGC in *B. neobassiana*, suggests that gene rearrangements and adaptive evolution of secondary metabolism may promote chemical diversity contributing to higher virulence. In addition, two orthogroups for chitinases are likely fast-evolving genes based on significant aBSREL tests (OG0003769 and OG0008694, Table S10) in some high-virulence strains. These candidates are promising to test whether enhanced expression of these genes can promote fungal virulence in insect hosts. Overall, our study supports chitinases and NRPS toxins as two of the most common molecular arsenals driving virulence in *Beauveria*, consistent with other entomopathogenic fungi [8,10, 54]. Studies by Zhang *et al*. and Gasmi *et al*. [21,22] previously identified positive selection acting on NRPS and chitinase genes, respectively. However, our approach differs in two key aspects. First, whereas both studies utilized genome-wide screens to broadly identify candidate genes, we utilized a targeted approach to specifically focus on orthogroups encoding chitinase and core NRPS biosynthetic genes. Second, our study encompasses a broader phylogenetic scale at the multispecies level, while these earlier studies targeted sequence polymorphism within a single species (intraspecific inference). Intraspecific datasets often capture limited genetic divergence, which can constrain the detection of positive selection that acts more strongly across evolutionary lineages.

### Independent evolution: interplay between gene gain/loss, rearrangement and positive selection

The inference of gene gain/loss events revealed frequent gene gains in high-virulence strains. Notably, *B. bassiana* NHJ13051 and *B. thailandica* MY4824 exhibited pronounced gene gains at their terminal nodes, associated with shifts to higher virulence. Likewise, ancestral nodes of the highly virulent *B. neobassiana* also displayed substantial gene gains. Conversely, low-virulence strains are often accompanied by gene losses, implying that reduction or loss of certain functions may hinder virulence.

Several well-known cases illustrate how gene gain/loss events underpin the acquisition or loss of virulence, particularly for fungal plant pathogens [92,94]. In entomopathogenic fungi, for instance, *Metarhizium anisopliae sensu lato* displays gain/loss events involving destruxin (non-ribosomal cyclopeptide toxins), differentiating between specialist and non-specialist species [95]. Our targeted heuristic analysis only identified one SMBGC highly associated with high virulence, putatively responsible for synthesizing a ‘deoxynivalenol-like’ compound. Deoxynivalenol, a trichothecene, typically has characteristics of both terpene and polyketide and is well-characterized among fungal plant pathogens [96]. Interestingly, the *Beauveria* ‘deoxynivalenol-like’ SMBGC identified in our study differs, comprising core genes for terpene and non-ribosomal peptide synthesis (instead of polyketide), suggesting the product might be a hybrid or entirely novel compound distinct from true trichothecenes. Its consistent presence across almost all the high-virulence strains except *B. thailandica* MY4824 indicates this strain might employ an alternative mechanism for virulence such as hydrolytic enzymes targeting cuticles. Furthermore, two variants of this SMBGC exist, defined by two separate orthogroups of terpene core genes, distinguishing *B. neobassiana* from *B. namnaoensis* and *B. bassiana*. This pattern suggests diversification of secondary metabolites potentially associated with fungal virulence, like patterns observed in *Tolypocladium inflatum* [97]. Future metabolite profiling and characterization will be beneficial for discovering novel compounds that could be used for pest insect control.

*B. bassiana* NHJ13051 and *B. thailandica* MY4824 also displayed the highest numbers of genes under positive selection among the high-virulence strains. In effect, positively selected genes are predominantly strain-specific, suggesting independent adaptive evolution. This adaptive evolution may explain intra- and interspecific variation in virulence. There are numerous examples of independent adaptive evolution in fungal plant pathogens explained by different types of molecular variation, e.g. distinct loci associated with virulence from different populations of the wheat pathogen *Zymoseptoria tritici* across the globe [94] and independent acquisition of entire pathogenicity chromosomes between genetically distinct lineages of *Fusarium oxysporum* [98]. Regarding entomopathogenic fungi, there have been limited accounts of molecular variation underlying virulence and host specificity from independent lineages. For *Metarhizium*, most of the efforts are concentrated around *M. anisopliae sensu lato* and closely related taxa where generalist species were characterized by the acquisition of gene clusters producing destruxins [74]. In *Beauveria*, studies focused on genomic features distinct between high- and low-virulence strains within species (intraspecific variation) toward given insects, e.g. mosquitoes [20], but, to our knowledge, there seems to be no study showcasing adaptive evolution underlying the virulence among *Beauveria* strains of different genetic backgrounds. Our study highlights a similar tendency for ‘virulence’ to evolve independently in parallel across different species, reinforcing that fungal pathogens commonly adapt through a combination of gene gain/loss, rearrangement and positive selection [8,10, 21, 22, 54].

### Future improvements

While our study provided novel insights into virulence evolution among *Beauveria* species, a few limitations need to be considered to further improve analyses. First, high-contiguity genome drafts are essential for more comprehensive analyses of the genomic signature underlying virulence. Although our sequencing data resulted in draft genomes covering most protein-coding regions (BUSCO completeness >98.0%, Table S1), higher-quality assemblies from long-read sequencing and transcriptome sequencing will facilitate more detailed comparative analyses such as synteny, repetitive elements and regulatory elements. Another limitation is that our virulence assays were conducted on a single host species, *S. exigua*, and that the findings on high/low virulence should not be generalized to all types of insect hosts. A recent study by Liu *et al*. [99] demonstrated that *B. bassiana* has different virulence levels (measured by insect mortality) when infecting three insect hosts: *Bombyx mori*, *Helicoverpa armigera* and *Clanis bilineata*. This study also revealed distinct gene-expression profiles when *B. bassiana* was inoculated on different insect hosts. Therefore, comparative pathogenicity analyses across multiple insect hosts would provide deeper insights into host specificity among different *Beauveria* species and clarify general versus host-specific virulence factors. Ultimately, integrating comparative transcriptomics with functional gene characterization will further elucidate the diversity of fungal virulence across multiple *Beauveria* species as well as their underlying mechanisms.

## Supplementary material

10.1099/mgen.0.001758 Supplementary Material 1.

10.1099/mgen.0.001758 Supplementary Material 2.

## Abbreviations

aBSREL: adaptive branch-site random effect likelihood
CAZyme: carbohydrate-active enzyme
GLM: generalized linear model
MFS: major facilitator superfamily
ML: maximum likelihood
NRPS: non-ribosomal peptide synthase
PDA: potato dextrose agar
PKS: polyketide synthase
PMS: profile match score
SMBGC: secondary metabolite biosynthetic gene cluster
VPS: virulence pair score
