# Supplementary material for: Gene presence–absence and evolutionary signals of positive selection associated with virulence divergence in Beauveria fungi
Source: Microb Genom. 2026 Jun 26;12(6):001758. doi: 10.1099/mgen.0.001758 (PMC13313595; doi:10.1099/mgen.0.001758)
Supplement: Supplementary Material 1. [file mgen-12-01758-s001.pdf]

Supplementary Figures

Gene presence–absence and evolutionary signals of positive selection associated with virulence divergence in *Beauveria* fungi

Teeratas Kijpornyongpan[1,2], Nuntanat Arnamnart[2], Alongkorn Amnuaykanjanasin[2], Janet Jennifer Luangsa-ard[2] and Noppol Kobmoo[2]*

[1]Department of Botany and Plant Pathology, Purdue University, West Lafayette IN, USA

[2]National Center for Genetic Engineering and Biotechnology (BIOTEC), National Science and Technology Development Agency (NSTDA), Pathum Thani, Thailand

*Corresponding author: noppol.kob@biotec.or.th

Supplementary text 1 Genome statistics

Genome assembly size ranged from 31.1 Mb (*B. neobassiana* MY5147) to 38.9 Mb (*B. thailandica* MY3296), in the expected range of genom size for *Beauveria*. GC content vary between 44.8% (*B. thailandica* MY3296) and 52.2% (*B. namnaoensis* MY3957). The N50 values as well as sequencing depth were positively correlated with the number of raw reads used in the assembly, similarly to a correlation between coverage depth and N50 (Figure S1A – C). However, an exception was observed for *B. gryllotalpidicola* MY11210, which exhibited the highest coverage (126x) but the 11th-highest N50 value. This discrepancy might result from its high proportion of repetitive elements, in particular the notably high percentage of simple repeats (2.43%) compared to other genomes (Table S1), leading to fragmentation in genome assembly. Overall, this explains that the sequencing yield has direct impact on the quality of the genome. Nevertheless, despite various N50 values (39.6 kb–196.7 kb) and coverage depth (13x–126x), all 15 genome assemblies showed high BUSCO (98.2 – 99.6%), indicating excellent integrity of genomic DNA samples used for sequencing (Table 1).

Gene prediction and annotation of the 15 *Beauveria* de novo drafted genomes from this study, along with the genome of *B. neobassiana* BCC2660, identified between 9,728 (*B. gryllotalpidicola* MY11210) and 10,377 (*B. bassiana* MY4038) genes per genome. A plot between assembly N50 value and a number of predicted genes shows a weakly positive correlation (Figure S1D, Pearson's r = 0.528, p-value = 0.043), and little variability explained by N50 value (adjusted R-squared = 0.223). There was no significant correlation between the virulence and the number of predicted genes (Figure S1E – F, Pearson's r between Mortality (%) Day7 and predicted gene number = 0.200, p-value = 0.475; Pearson's r between Mycelial colonization (%) Day7 and predicted gene number = 0.106, p-value = 0.706), suggesting no bias for using gene presence–absence data to detect genes associated with virulence. The gene length, measured from translation start to termination sites, was averaged 1,600 bp for all 16 genomes with an average exon count ranging between 2.54 – 2.59 and an average intron length of approximately 100 bp. Notably, two genomes of *B. thailandica* MY3296, MY4824 had longer average

intron lengths, measuring 145 and 123 bp, respectively (Table S1). Most of the protein-coding genes have functional annotation from eggNOG mapper (93.5% in average) and/or protein domain annotation from InterproScan (92.0% in average). However, *B. asiatica* ARSEF4850 displayed lower annotation completeness, with only 88.0% of genes annotated via eggNOG mapper and 86.8% via InterproScan, respectively (Table S1).

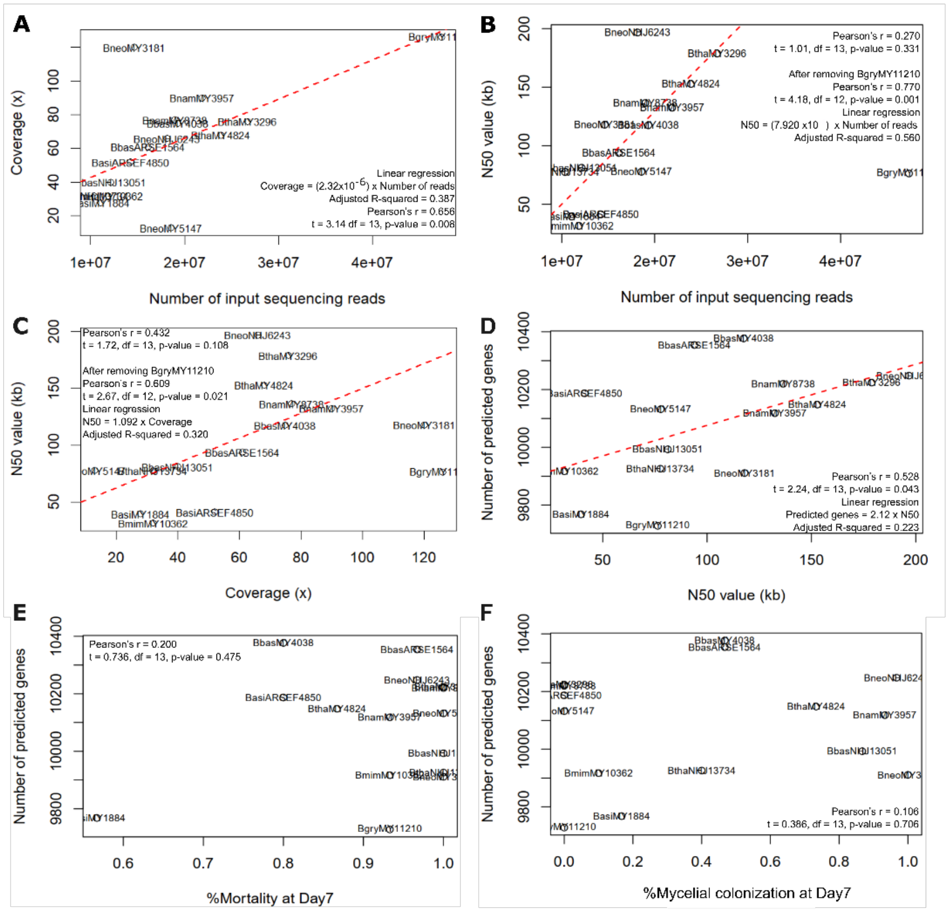

Figure S1 Scatterplots showing relationships between numbers of input DNA sequencing reads for assembly, sequencing coverage, and N_{50} values of genome assembly. (A) Scatterplot between input DNA sequencing reads and coverage. (B) Scatterplot between input DNA sequencing reads and N_{50} value. (C) Scatterplot between coverage and N_{50} value. (D) Scatterplot between N_{50} value and number of predicted genes. (E and F) Scatterplots between virulence (Mortality % in E and Mycelial colonization % in F) and number of predicted genes. Each dot, attached with a strain abbreviation, indicates a data point of each genome assembly. Pearson's correlation is calculated to test relationships between two variables. Linear regression lines are drawn for each plot to see the data trend between each pair of variables. Note that the assembly of *B. gryllotalpidicola* MY11210 has an extreme value that could not align with the other assemblies. We therefore remove MY11210 from the dataset and reanalyze the Pearson's correlations for panels (B) and (C).

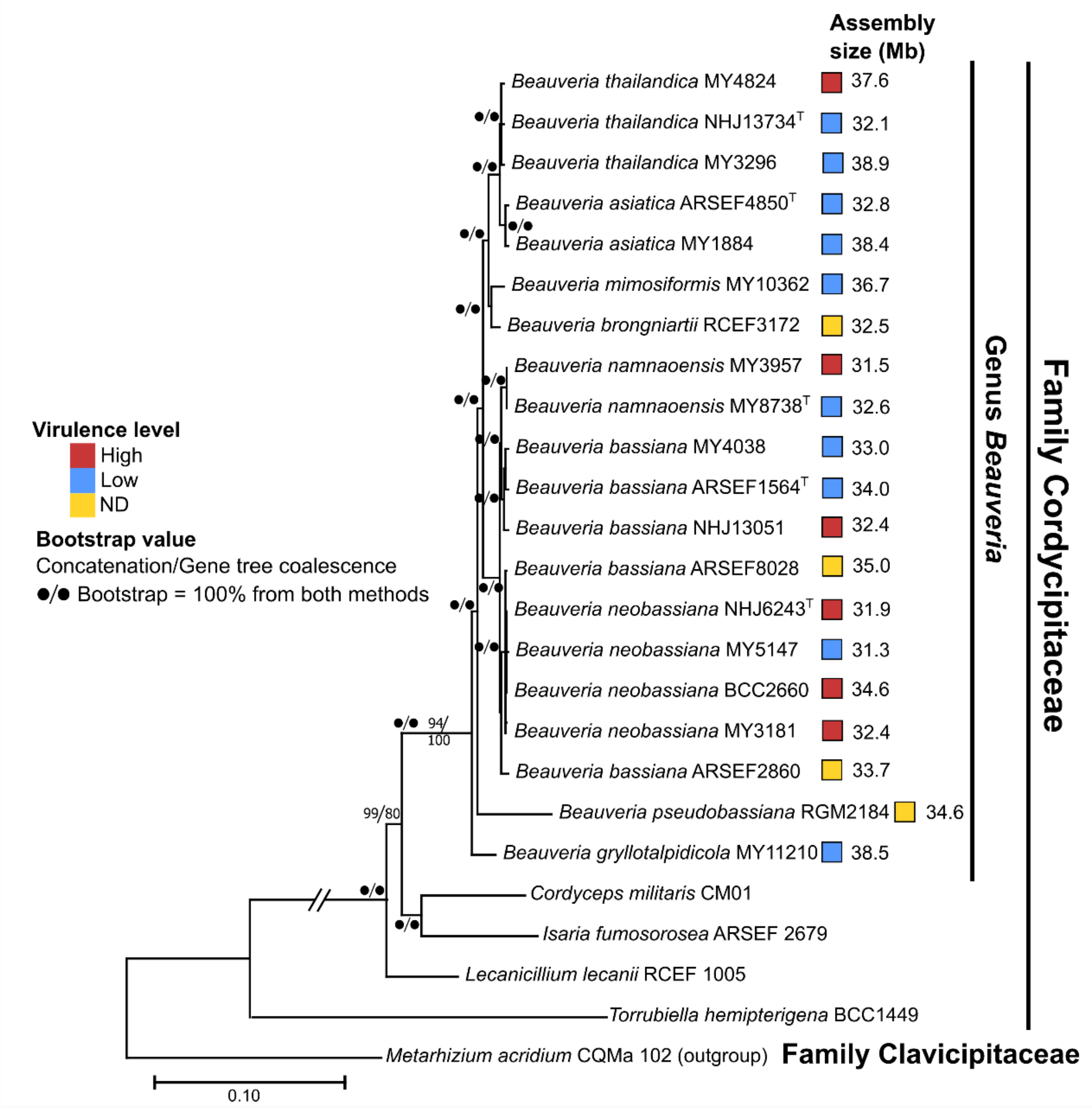

Figure S2 Phylogenomic species tree of seventeen *Beauveria* strains, and other affiliated *Beauveria* species. Sixteen *Beauveria* genomes generated in this study, plus additional *Beauveria* genomes and representative genomes from Cordycipitaceae, were included in the analyses. The species trees reconstructed from two methods (concatenation-maximum likelihood and gene tree-species tree coalescence) yield identical topology. Circle symbols on nodes indicate 100% bootstrap values from each method, separated by a backslash. Legends after a taxon name show virulence level and the genome assembly size.

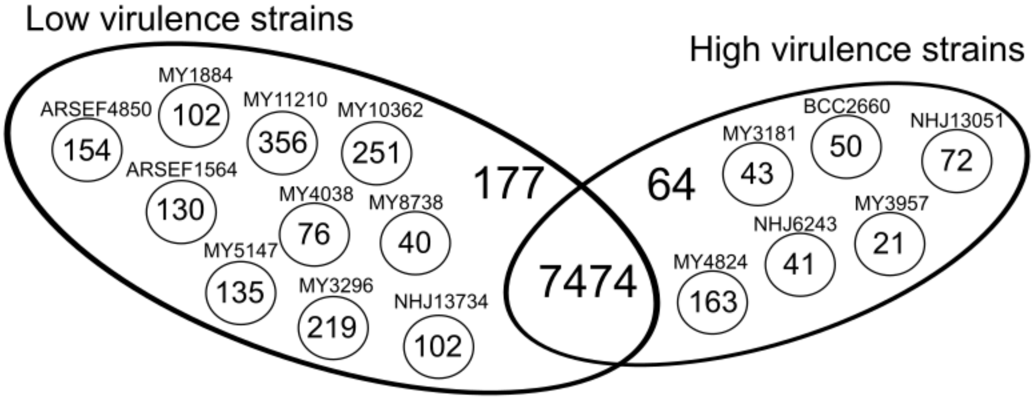

Figure S3 The Venn diagram shows the distribution of orthogroups among 16 *Beauveria* strains. Numbers in small circles indicate orthogroups present uniquely in a single genome (strain-specific). Numbers in the non-overlapping areas of each oval represent orthogroups shared by at least two strains within either the low- or high-virulence groups, but absent from the other group. The intersected area of the ovals indicates orthogroups shared by all 16 genomes.

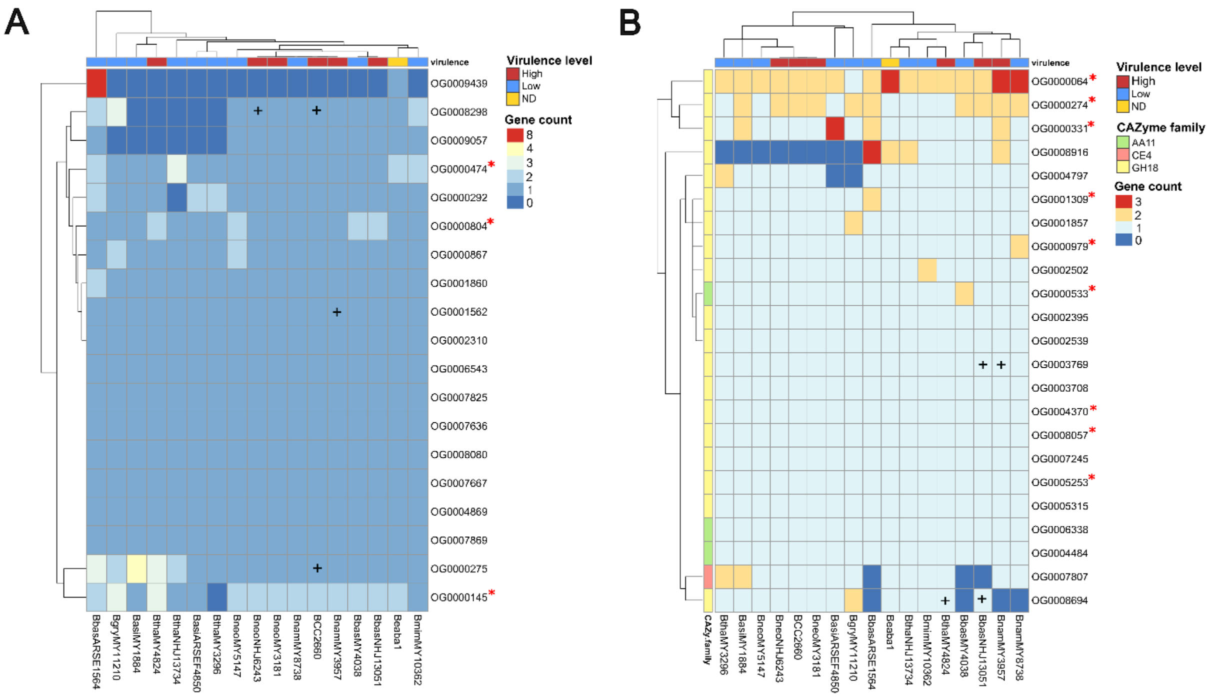

Figure S4 Targeted comparative genomics for virulence-associated genes among 17 *Beauveria* genomes. (A) Heatmap showing the distribution of NRPS core biosynthetic genes in *B. bassiana* ARSEF2860 and respective orthologs in the other genomes. NRPS core genes with known synthesized secondary metabolites are labelled as texts below gene IDs. (B) Heatmap showing the distribution of chitin-specific CAZyme genes in *B. bassiana* ARSEF2860 and respective orthologs in the other genomes. Color keys at the right side of *B. bassiana* ARSEF2860 gene IDs indicate types of CAZyme families. For (A) and (B), row clustering is based on phylogenetic relationships of *B. bassiana* ARSEF2860 genes determined by a Neighbor-joining tree. Plus signs (+) in cells indicate orthologs that are under positive diversifying selection based on significant aBSREL tests in high-virulence strains. Red asterisks indicate that are overexpressed in the high-virulence strain of *B. bassiana* GXsk1011 compared to the low-virulence strain GXtr1009 when both strains were exposed with insect cuticular extract (Wang et al., 2017)

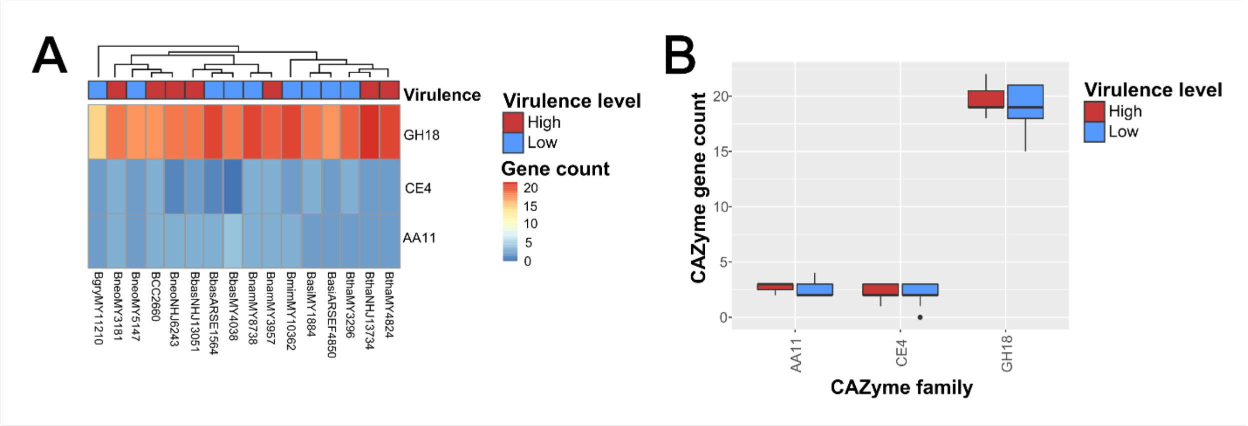

Figure S5 Distribution of genes encoding chitin-specific CAZymes across 16 *Beauveria* genomes. (A) A heatmap showing the distribution of three chitin-specific CAZyme families: glycoside hydrolase family 18 (GH18), carbohydrate esterase family 4 (CE4), and auxiliary activity family 11 (AA11). Columns indicate strain abbreviations for 16 genomes. Heatmap colors in cells indicate gene count. The heatmap is clustered based on a Euclidean distance. (B) A boxplot showing the distribution of chitin-specific CAZymes between genomes of the fungal isolates having high and low pathogenicity. No statistical difference between two types of pathogenicity for all CAZyme families.
